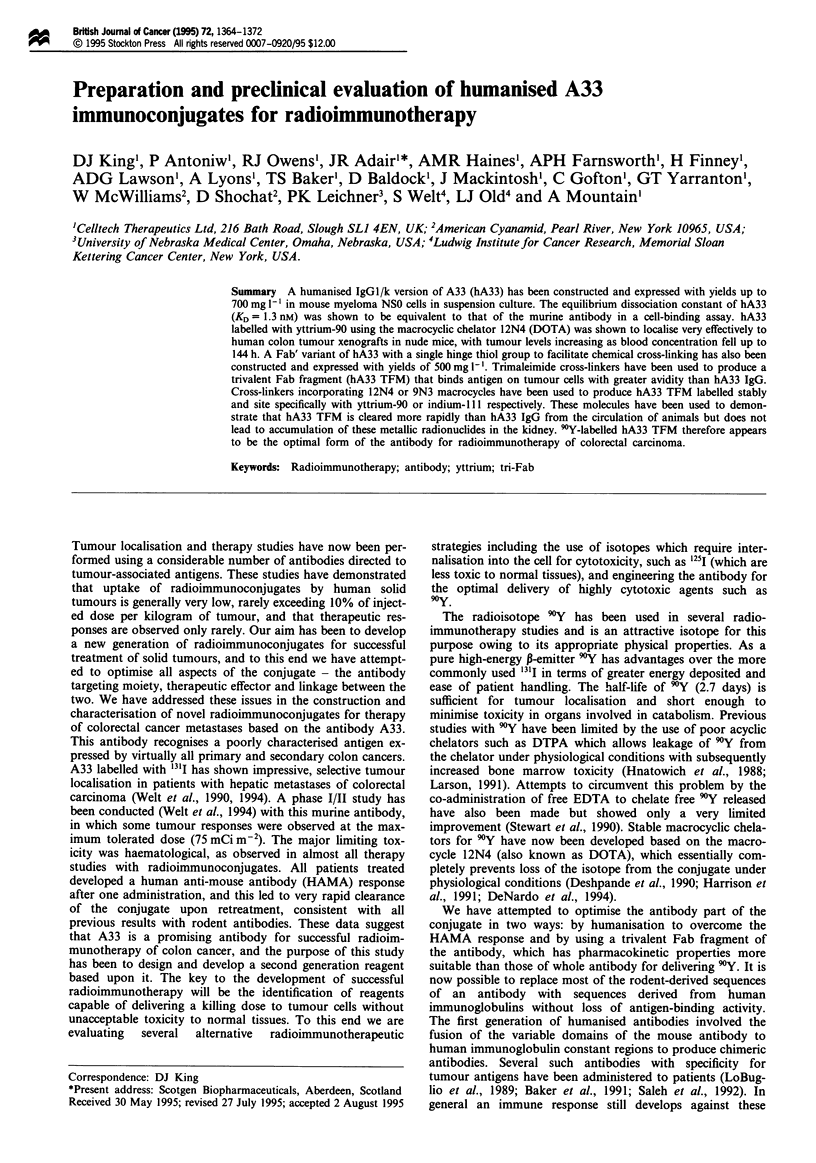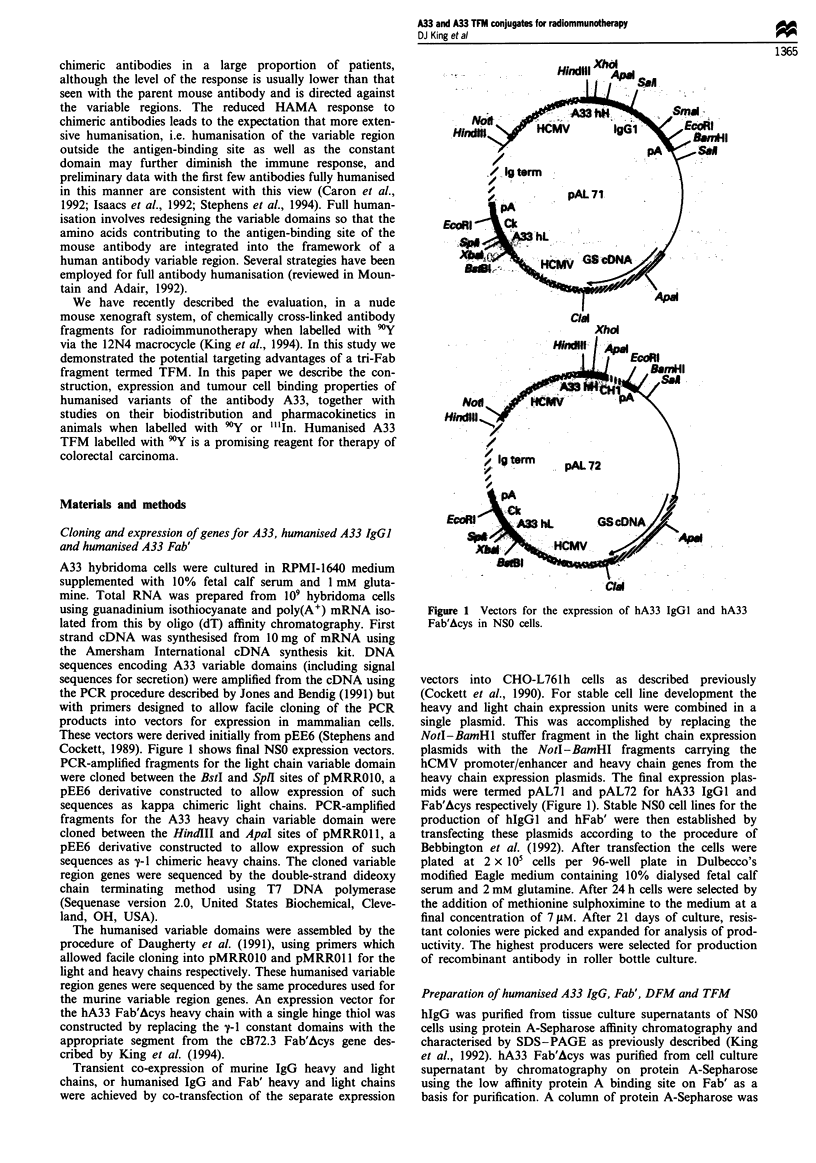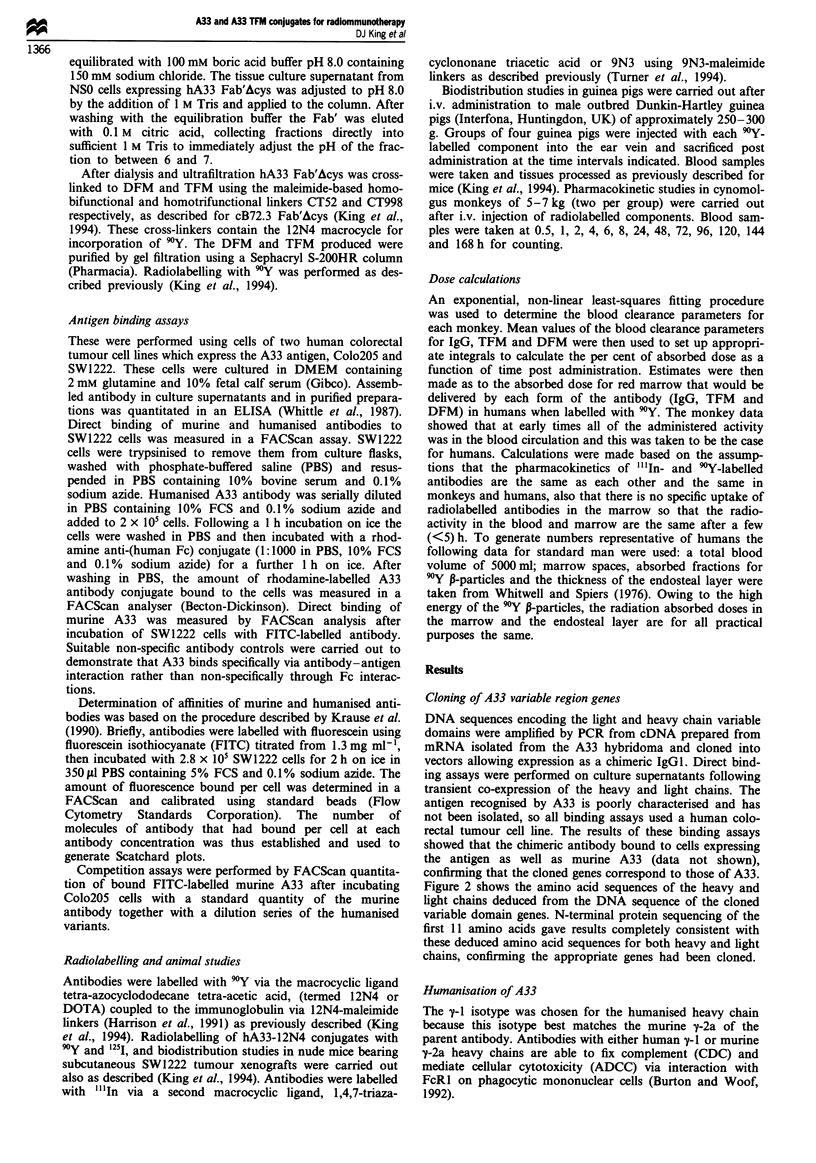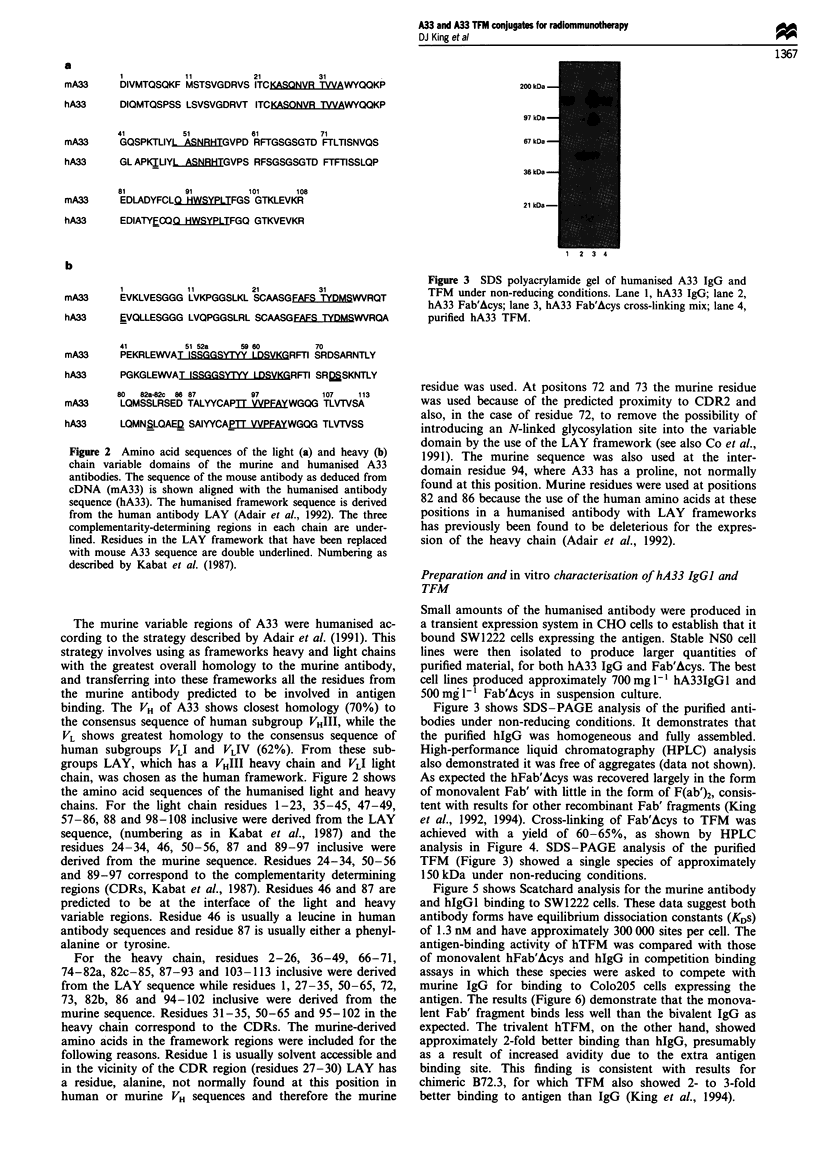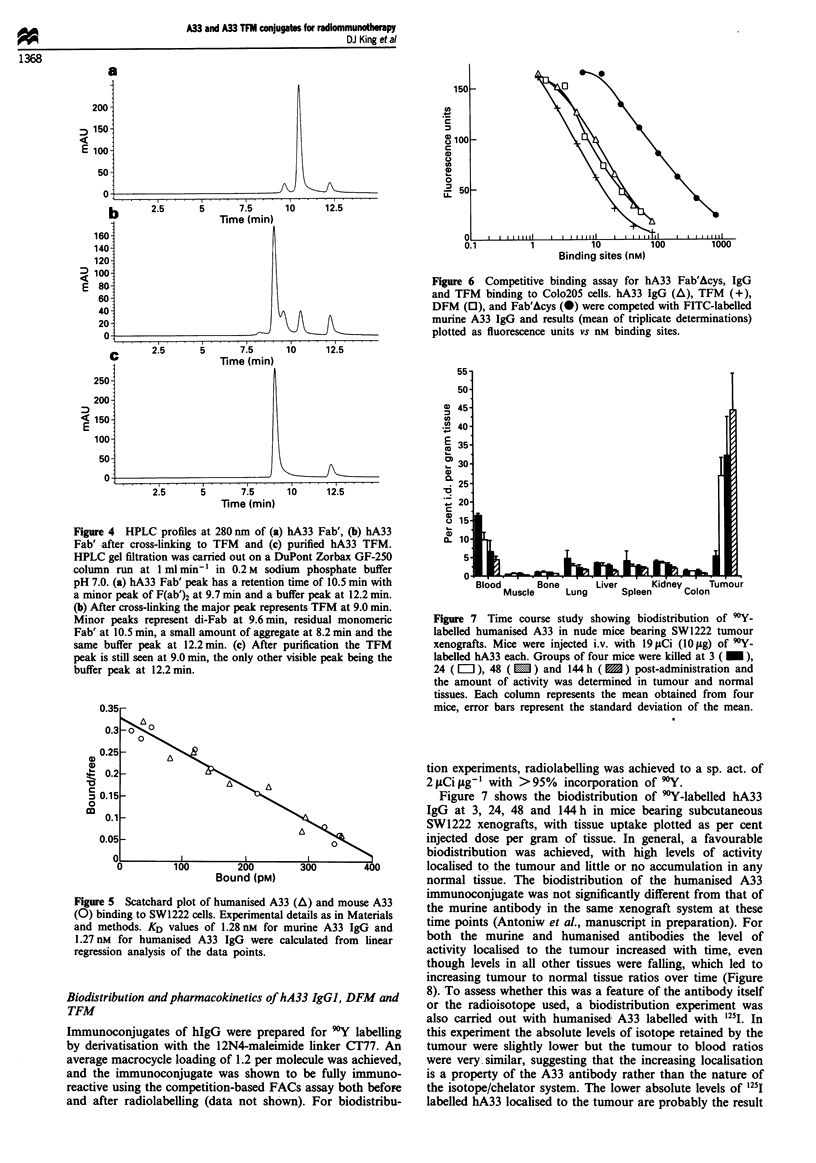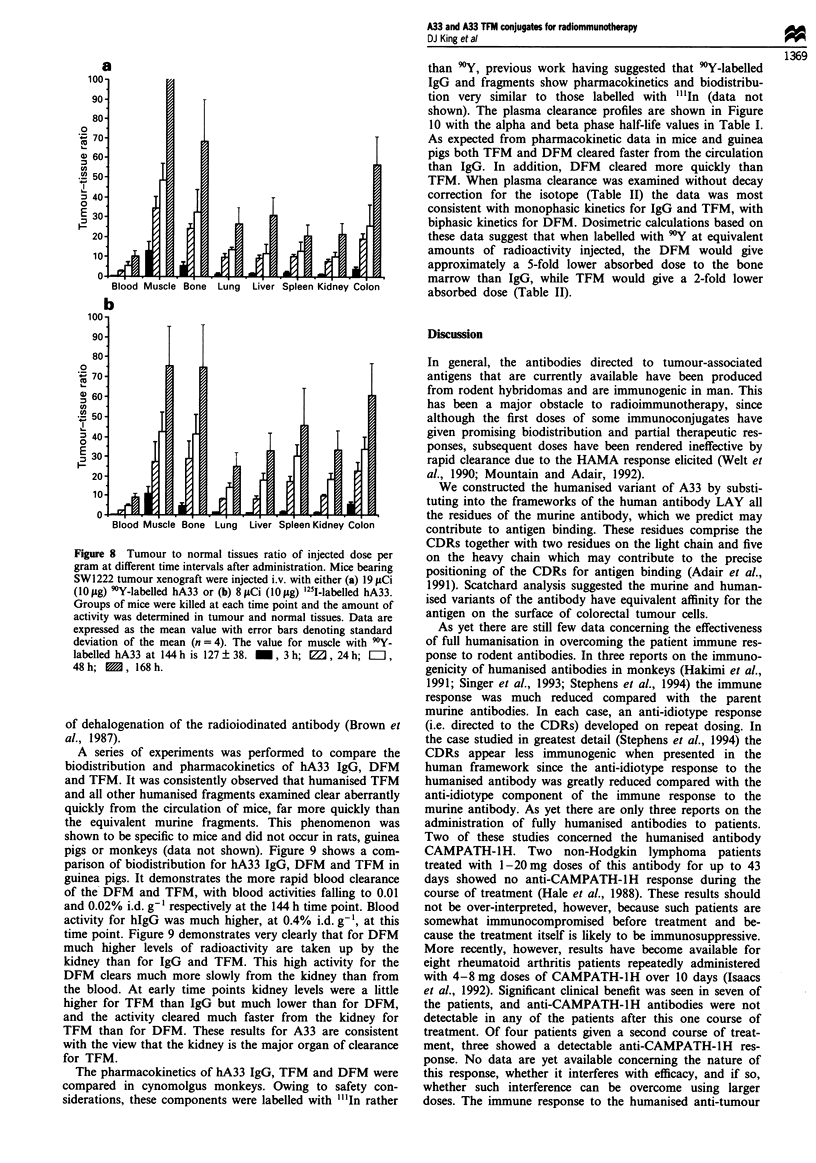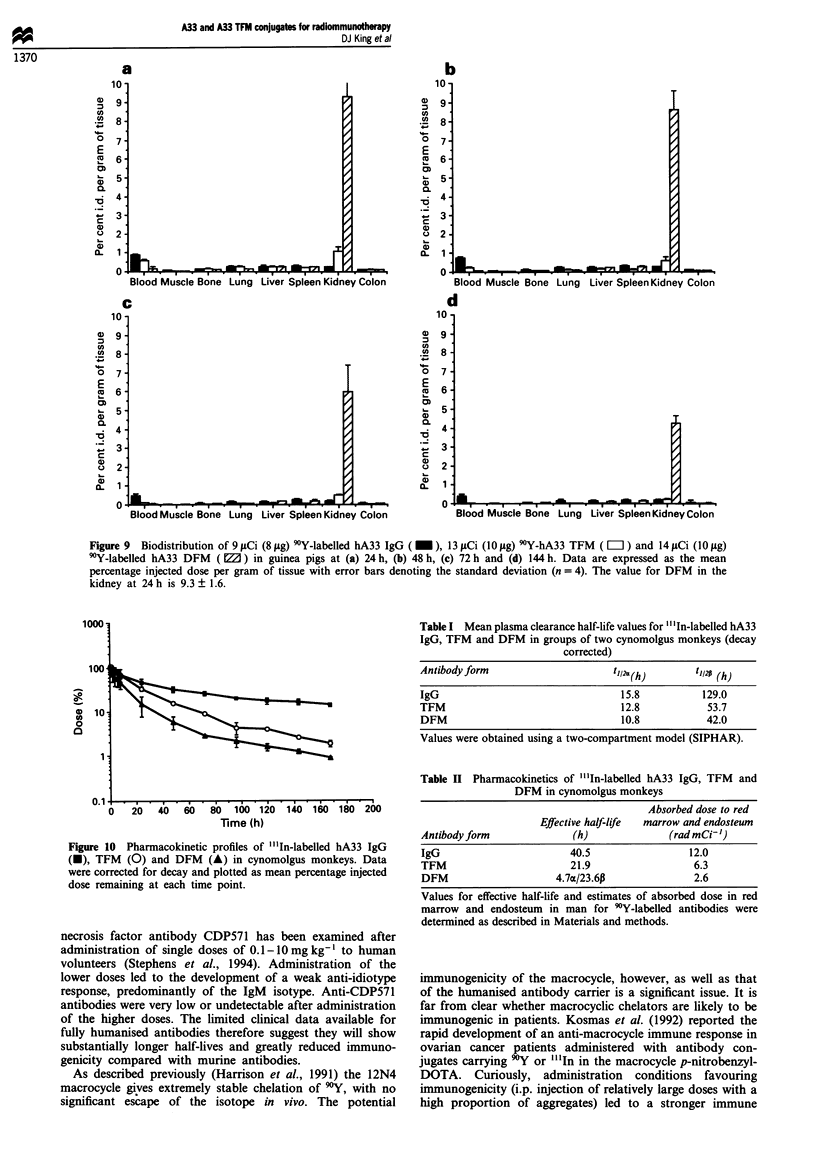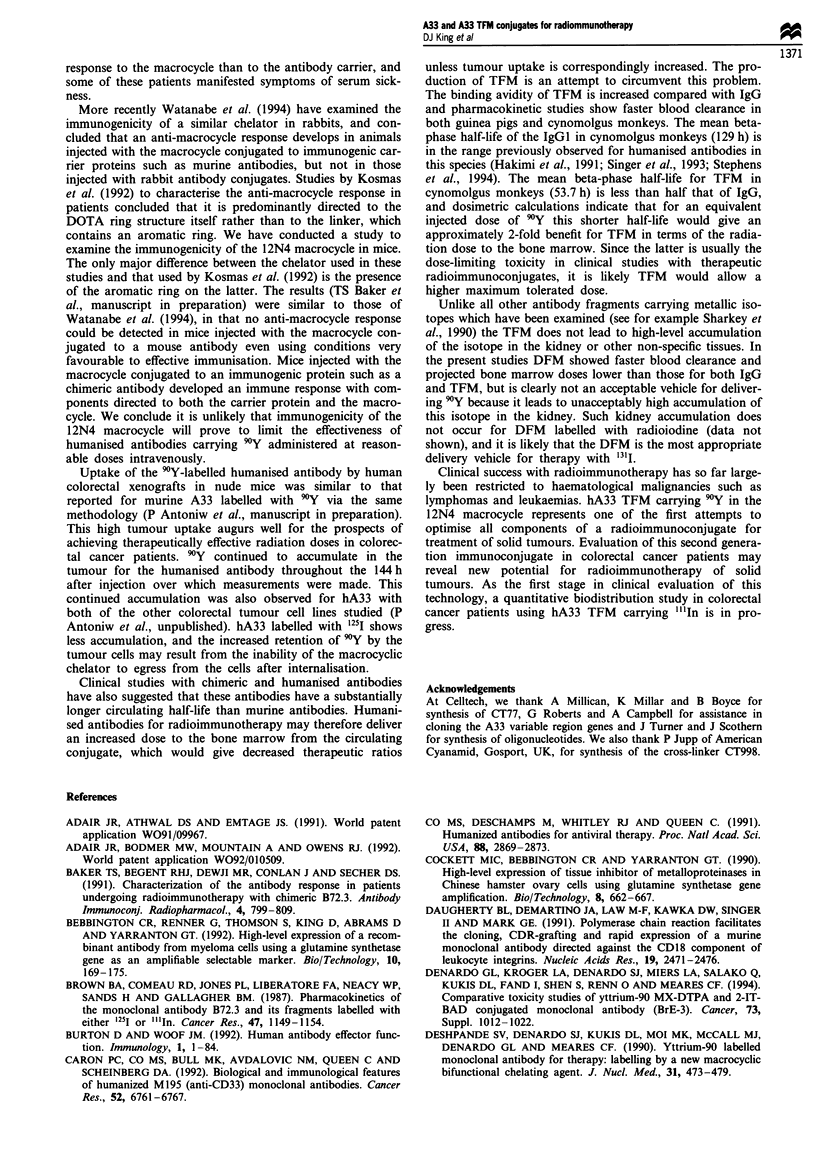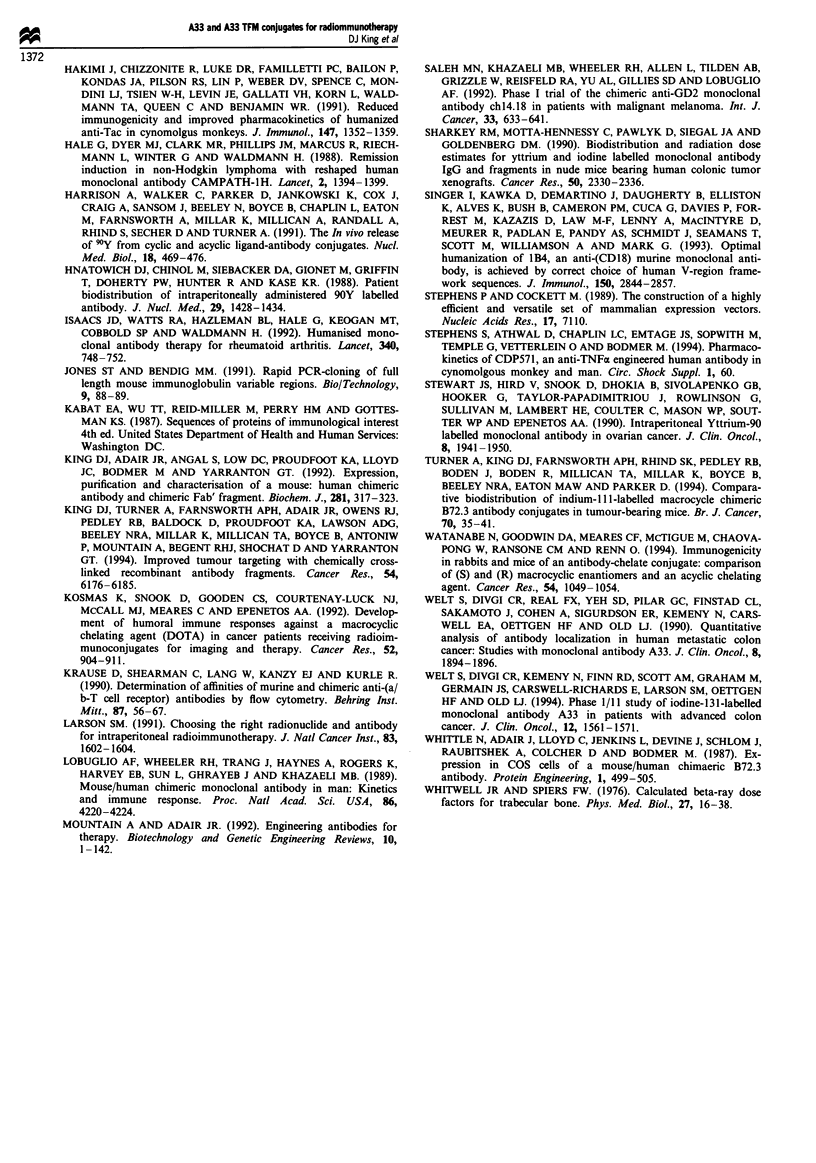# Preparation and preclinical evaluation of humanised A33 immunoconjugates for radioimmunotherapy.

**DOI:** 10.1038/bjc.1995.516

**Published:** 1995-12

**Authors:** D. J. King, P. Antoniw, R. J. Owens, J. R. Adair, A. M. Haines, A. P. Farnsworth, H. Finney, A. D. Lawson, A. Lyons, T. S. Baker

**Affiliations:** Celltech Therapeutics Ltd., Slough, UK.

## Abstract

**Images:**


British Journal of Cancer (1995) 72, 1364–1372
© 1995 Stockton Press All rights reserved 0007-0920/95 $12.00

### Preparation and preclinical evaluation of humanised A33 immunoconjugates for radioimmunotherapy

DJ King[1], P Antoniw[1], RJ Owens[1], JR Adair[1]*, AMR Haines[1], APH Farnsworth[1], H Finney[1], ADG Lawson[1], A Lyons[1], TS Baker[1], D Baldock[1], J Mackintosh[1], C Gofton[1], GT Yarranton[1], W McWilliams[2], D Shochat[2], PK Leichner[3], S Welt[4], LJ Old[4] and A Mountain[1]

[1]*Celltech Therapeutics Ltd, 216 Bath Road, Slough SL1 4EN, UK;* [2]*American Cyanamid, Pearl River, New York 10965, USA;* [3]*University of Nebraska Medical Center, Omaha, Nebraska, USA;* [4]*Ludwig Institute for Cancer Research, Memorial Sloan Kettering Cancer Center, New York, USA.*

**Summary** A humanised IgG1/k version of A33 (hA33) has been constructed and expressed with yields up to 700 mg $l^{-1}$ in mouse myeloma NS0 cells in suspension culture. The equilibrium dissociation constant of hA33 ($K_D$ = 1.3 nM) was shown to be equivalent to that of the murine antibody in a cell-binding assay. hA33 labelled with yttrium-90 using the macrocyclic chelator 12N4 (DOTA) was shown to localise very effectively to human colon tumour xenografts in nude mice, with tumour levels increasing as blood concentration fell up to 144 h. A Fab′ variant of hA33 with a single hinge thiol group to facilitate chemical cross-linking has also been constructed and expressed with yields of 500 mg $l^{-1}$. Trimaleimide cross-linkers have been used to produce a trivalent Fab fragment (hA33 TFM) that binds antigen on tumour cells with greater avidity than hA33 IgG. Cross-linkers incorporating 12N4 or 9N3 macrocycles have been used to produce hA33 TFM labelled stably and site specifically with yttrium-90 or indium-111 respectively. These molecules have been used to demonstrate that hA33 TFM is cleared more rapidly than hA33 IgG from the circulation of animals but does not lead to accumulation of these metallic radionuclides in the kidney. $^{90}$Y-labelled hA33 TFM therefore appears to be the optimal form of the antibody for radioimmunotherapy of colorectal carcinoma.

**Keywords:** Radioimmunotherapy; antibody; yttrium; tri-Fab

Tumour localisation and therapy studies have now been performed using a considerable number of antibodies directed to tumour-associated antigens. These studies have demonstrated that uptake of radioimmunoconjugates by human solid tumours is generally very low, rarely exceeding 10% of injected dose per kilogram of tumour, and that therapeutic responses are observed only rarely. Our aim has been to develop a new generation of radioimmunoconjugates for successful treatment of solid tumours, and to this end we have attempted to optimise all aspects of the conjugate – the antibody targeting moiety, therapeutic effector and linkage between the two. We have addressed these issues in the construction and characterisation of novel radioimmunoconjugates for therapy of colorectal cancer metastases based on the antibody A33. This antibody recognises a poorly characterised antigen expressed by virtually all primary and secondary colon cancers. A33 labelled with $^{131}$I has shown impressive, selective tumour localisation in patients with hepatic metastases of colorectal carcinoma (Welt *et al.*, 1990, 1994). A phase I/II study has been conducted (Welt *et al.*, 1994) with this murine antibody, in which some tumour responses were observed at the maximum tolerated dose (75 mCi $m^{-2}$). The major limiting toxicity was haematological, as observed in almost all therapy studies with radioimmunoconjugates. All patients treated developed a human anti-mouse antibody (HAMA) response after one administration, and this led to very rapid clearance of the conjugate upon retreatment, consistent with all previous results with rodent antibodies. These data suggest that A33 is a promising antibody for successful radioimmunotherapy of colon cancer, and the purpose of this study has been to design and develop a second generation reagent based upon it. The key to the development of successful radioimmunotherapy will be the identification of reagents capable of delivering a killing dose to tumour cells without unacceptable toxicity to normal tissues. To this end we are evaluating several alternative radioimmunotherapeutic strategies including the use of isotopes which require internalisation into the cell for cytotoxicity, such as $^{125}$I (which are less toxic to normal tissues), and engineering the antibody for the optimal delivery of highly cytotoxic agents such as $^{90}$Y.

The radioisotope $^{90}$Y has been used in several radioimmunotherapy studies and is an attractive isotope for this purpose owing to its appropriate physical properties. As a pure high-energy $\beta$-emitter $^{90}$Y has advantages over the more commonly used $^{131}$I in terms of greater energy deposited and ease of patient handling. The half-life of $^{90}$Y (2.7 days) is sufficient for tumour localisation and short enough to minimise toxicity in organs involved in catabolism. Previous studies with $^{90}$Y have been limited by the use of poor acyclic chelators such as DTPA which allows leakage of $^{90}$Y from the chelator under physiological conditions with subsequently increased bone marrow toxicity (Hnatowich *et al.*, 1988; Larson, 1991). Attempts to circumvent this problem by the co-administration of free EDTA to chelate free $^{90}$Y released have also been made but showed only a very limited improvement (Stewart *et al.*, 1990). Stable macrocyclic chelators for $^{90}$Y have now been developed based on the macrocycle 12N4 (also known as DOTA), which essentially completely prevents loss of the isotope from the conjugate under physiological conditions (Deshpande *et al.*, 1990; Harrison *et al.*, 1991; DeNardo *et al.*, 1994).

We have attempted to optimise the antibody part of the conjugate in two ways: by humanisation to overcome the HAMA response and by using a trivalent Fab fragment of the antibody, which has pharmacokinetic properties more suitable than those of whole antibody for delivering $^{90}$Y. It is now possible to replace most of the rodent-derived sequences of an antibody with sequences derived from human immunoglobulins without loss of antigen-binding activity. The first generation of humanised antibodies involved the fusion of the variable domains of the mouse antibody to human immunoglobulin constant regions to produce chimeric antibodies. Several such antibodies with specificity for tumour antigens have been administered to patients (LoBuglio *et al.*, 1989; Baker *et al.*, 1991; Saleh *et al.*, 1992). In general an immune response still develops against these

Correspondence: DJ King
*Present address: Scotgen Biopharmaceuticals, Aberdeen, Scotland
Received 30 May 1995; revised 27 July 1995; accepted 2 August 1995

chimeric antibodies in a large proportion of patients, although the level of the response is usually lower than that seen with the parent mouse antibody and is directed against the variable regions. The reduced HAMA response to chimeric antibodies leads to the expectation that more extensive humanisation, i.e. humanisation of the variable region outside the antigen-binding site as well as the constant domain may further diminish the immune response, and preliminary data with the first few antibodies fully humanised in this manner are consistent with this view (Caron *et al.*, 1992; Isaacs *et al.*, 1992; Stephens *et al.*, 1994). Full humanisation involves redesigning the variable domains so that the amino acids contributing to the antigen-binding site of the mouse antibody are integrated into the framework of a human antibody variable region. Several strategies have been employed for full antibody humanisation (reviewed in Mountain and Adair, 1992).

We have recently described the evaluation, in a nude mouse xenograft system, of chemically cross-linked antibody fragments for radioimmunotherapy when labelled with $^{90}$Y via the 12N4 macrocycle (King *et al.*, 1994). In this study we demonstrated the potential targeting advantages of a tri-Fab fragment termed TFM. In this paper we describe the construction, expression and tumour cell binding properties of humanised variants of the antibody A33, together with studies on their biodistribution and pharmacokinetics in animals when labelled with $^{90}$Y or $^{111}$In. Humanised A33 TFM labelled with $^{90}$Y is a promising reagent for therapy of colorectal carcinoma.

### Materials and methods

### *Cloning and expression of genes for A33, humanised A33 IgG1 and humanised A33 Fab'*

A33 hybridoma cells were cultured in RPMI-1640 medium supplemented with 10% fetal calf serum and 1 mM glutamine. Total RNA was prepared from $10^9$ hybridoma cells using guanadinium isothiocyanate and poly($A^+$) mRNA isolated from this by oligo (dT) affinity chromatography. First strand cDNA was synthesised from 10 mg of mRNA using the Amersham International cDNA synthesis kit. DNA sequences encoding A33 variable domains (including signal sequences for secretion) were amplified from the cDNA using the PCR procedure described by Jones and Bendig (1991) but with primers designed to allow facile cloning of the PCR products into vectors for expression in mammalian cells. These vectors were derived initially from pEE6 (Stephens and Cockett, 1989). Figure 1 shows final NSO expression vectors. PCR-amplified fragments for the light chain variable domain were cloned between the *Bst*I and *Spl*I sites of pMRR010, a pEE6 derivative constructed to allow expression of such sequences as kappa chimeric light chains. PCR-amplified fragments for the A33 heavy chain variable domain were cloned between the *Hind*III and *Apa*I sites of pMRR011, a pEE6 derivative constructed to allow expression of such sequences as γ-1 chimeric heavy chains. The cloned variable region genes were sequenced by the double-strand dideoxy chain terminating method using T7 DNA polymerase (Sequenase version 2.0, United States Biochemical, Cleveland, OH, USA).

The humanised variable domains were assembled by the procedure of Daugherty *et al.* (1991), using primers which allowed facile cloning into pMRR010 and pMRR011 for the light and heavy chains respectively. These humanised variable region genes were sequenced by the same procedures used for the murine variable region genes. An expression vector for the hA33 Fab'Δcys heavy chain with a single hinge thiol was constructed by replacing the γ-1 constant domains with the appropriate segment from the cB72.3 Fab'Δcys gene described by King *et al.* (1994).

Transient co-expression of murine IgG heavy and light chains, or humanised IgG and Fab' heavy and light chains were achieved by co-transfection of the separate expression

**Figure 1** Vectors for the expression of hA33 IgG1 and hA33 Fab'Δcys in NSO cells.

vectors into CHO-L761h cells as described previously (Cockett *et al.*, 1990). For stable cell line development the heavy and light chain expression units were combined in a single plasmid. This was accomplished by replacing the *Not*I–*Bam*HI stuffer fragment in the light chain expression plasmids with the *Not*I–*Bam*HI fragments carrying the hCMV promoter/enhancer and heavy chain genes from the heavy chain expression plasmids. The final expression plasmids were termed pAL71 and pAL72 for hA33 IgG1 and Fab'Δcys respectively (Figure 1). Stable NSO cell lines for the production of hIgG1 and hFab' were then established by transfecting these plasmids according to the procedure of Bebbington *et al.* (1992). After transfection the cells were plated at $2 \times 10^5$ cells per 96-well plate in Dulbecco's modified Eagle medium containing 10% dialysed fetal calf serum and 2 mM glutamine. After 24 h cells were selected by the addition of methionine sulphoximine to the medium at a final concentration of 7 μM. After 21 days of culture, resistant colonies were picked and expanded for analysis of productivity. The highest producers were selected for production of recombinant antibody in roller bottle culture.

### *Preparation of humanised A33 IgG, Fab', DFM and TFM*

hIgG was purified from tissue culture supernatants of NSO cells using protein A-Sepharose affinity chromatography and characterised by SDS–PAGE as previously described (King *et al.*, 1992). hA33 Fab'Δcys was purified from cell culture supernatant by chromatography on protein A-Sepharose using the low affinity protein A binding site on Fab' as a basis for purification. A column of protein A-Sepharose was equilibrated with 100 mM boric acid buffer pH 8.0 containing 150 mM sodium chloride. The tissue culture supernatant from NS0 cells expressing hA33 Fab'Δcys was adjusted to pH 8.0 by the addition of 1 M Tris and applied to the column. After washing with the equilibration buffer the Fab' was eluted with 0.1 M citric acid, collecting fractions directly into sufficient 1 M Tris to immediately adjust the pH of the fraction to between 6 and 7.

After dialysis and ultrafiltration hA33 Fab'Δcys was cross-linked to DFM and TFM using the maleimide-based homobifunctional and homotrifunctional linkers CT52 and CT998 respectively, as described for cB72.3 Fab'Δcys (King *et al.*, 1994). These cross-linkers contain the 12N4 macrocycle for incorporation of $^{90}Y$. The DFM and TFM produced were purified by gel filtration using a Sephacryl S-200HR column (Pharmacia). Radiolabelling with $^{90}Y$ was performed as described previously (King *et al.*, 1994).

### *Antigen binding assays*

These were performed using cells of two human colorectal tumour cell lines which express the A33 antigen, Colo205 and SW1222. These cells were cultured in DMEM containing 2 mM glutamine and 10% fetal calf serum (Gibco). Assembled antibody in culture supernatants and in purified preparations was quantitated in an ELISA (Whittle *et al.*, 1987). Direct binding of murine and humanised antibodies to SW1222 cells was measured in a FACScan assay. SW1222 cells were trypsinised to remove them from culture flasks, washed with phosphate-buffered saline (PBS) and resuspended in PBS containing 10% bovine serum and 0.1% sodium azide. Humanised A33 antibody was serially diluted in PBS containing 10% FCS and 0.1% sodium azide and added to $2 \times 10^5$ cells. Following a 1 h incubation on ice the cells were washed in PBS and then incubated with a rhodamine anti-(human Fc) conjugate (1:1000 in PBS, 10% FCS and 0.1% sodium azide) for a further 1 h on ice. After washing in PBS, the amount of rhodamine-labelled A33 antibody conjugate bound to the cells was measured in a FACScan analyser (Becton-Dickinson). Direct binding of murine A33 was measured by FACScan analysis after incubation of SW1222 cells with FITC-labelled antibody. Suitable non-specific antibody controls were carried out to demonstrate that A33 binds specifically via antibody–antigen interaction rather than non-specifically through Fc interactions.

Determination of affinities of murine and humanised antibodies was based on the procedure described by Krause *et al.* (1990). Briefly, antibodies were labelled with fluorescein using fluorescein isothiocyanate (FITC) titrated from 1.3 mg $ml^{-1}$, then incubated with $2.8 \times 10^5$ SW1222 cells for 2 h on ice in 350 μl PBS containing 5% FCS and 0.1% sodium azide. The amount of fluorescence bound per cell was determined in a FACScan and calibrated using standard beads (Flow Cytometry Standards Corporation). The number of molecules of antibody that had bound per cell at each antibody concentration was thus established and used to generate Scatchard plots.

Competition assays were performed by FACScan quantitation of bound FITC-labelled murine A33 after incubating Colo205 cells with a standard quantity of the murine antibody together with a dilution series of the humanised variants.

### *Radiolabelling and animal studies*

Antibodies were labelled with $^{90}Y$ via the macrocyclic ligand tetra-azocyclododecane tetra-acetic acid, (termed 12N4 or DOTA) coupled to the immunoglobulin via 12N4-maleimide linkers (Harrison *et al.*, 1991) as previously described (King *et al.*, 1994). Radiolabelling of hA33-12N4 conjugates with $^{90}Y$ and $^{125}I$, and biodistribution studies in nude mice bearing subcutaneous SW1222 tumour xenografts were carried out also as described (King *et al.*, 1994). Antibodies were labelled with $^{111}In$ via a second macrocyclic ligand, 1,4,7-triazacyclononane triacetic acid or 9N3 using 9N3-maleimide linkers as described previously (Turner *et al.*, 1994).

Biodistribution studies in guinea pigs were carried out after i.v. administration to male outbred Dunkin-Hartley guinea pigs (Interfona, Huntingdon, UK) of approximately 250–300 g. Groups of four guinea pigs were injected with each $^{90}Y$-labelled component into the ear vein and sacrificed post administration at the time intervals indicated. Blood samples were taken and tissues processed as previously described for mice (King *et al.*, 1994). Pharmacokinetic studies in cynomolgus monkeys of 5–7 kg (two per group) were carried out after i.v. injection of radiolabelled components. Blood samples were taken at 0.5, 1, 2, 4, 6, 8, 24, 48, 72, 96, 120, 144 and 168 h for counting.

### *Dose calculations*

An exponential, non-linear least-squares fitting procedure was used to determine the blood clearance parameters for each monkey. Mean values of the blood clearance parameters for IgG, TFM and DFM were then used to set up appropriate integrals to calculate the per cent of absorbed dose as a function of time post administration. Estimates were then made as to the absorbed dose for red marrow that would be delivered by each form of the antibody (IgG, TFM and DFM) in humans when labelled with $^{90}Y$. The monkey data showed that at early times all of the administered activity was in the blood circulation and this was taken to be the case for humans. Calculations were made based on the assumptions that the pharmacokinetics of $^{111}In$- and $^{90}Y$-labelled antibodies are the same as each other and the same in monkeys and humans, also that there is no specific uptake of radiolabelled antibodies in the marrow so that the radioactivity in the blood and marrow are the same after a few (<5) h. To generate numbers representative of humans the following data for standard man were used: a total blood volume of 5000 ml; marrow spaces, absorbed fractions for $^{90}Y$ β-particles and the thickness of the endosteal layer were taken from Whitwell and Spiers (1976). Owing to the high energy of the $^{90}Y$ β-particles, the radiation absorbed doses in the marrow and the endosteal layer are for all practical purposes the same.

### Results

### *Cloning of A33 variable region genes*

DNA sequences encoding the light and heavy chain variable domains were amplified by PCR from cDNA prepared from mRNA isolated from the A33 hybridoma and cloned into vectors allowing expression as a chimeric IgG1. Direct binding assays were performed on culture supernatants following transient co-expression of the heavy and light chains. The antigen recognised by A33 is poorly characterised and has not been isolated, so all binding assays used a human colorectal tumour cell line. The results of these binding assays showed that the chimeric antibody bound to cells expressing the antigen as well as murine A33 (data not shown), confirming that the cloned genes correspond to those of A33. Figure 2 shows the amino acid sequences of the heavy and light chains deduced from the DNA sequence of the cloned variable domain genes. N-terminal protein sequencing of the first 11 amino acids gave results completely consistent with these deduced amino acid sequences for both heavy and light chains, confirming the appropriate genes had been cloned.

### *Humanisation of A33*

The γ-1 isotype was chosen for the humanised heavy chain because this isotype best matches the murine γ-2a of the parent antibody. Antibodies with either human γ-1 or murine γ-2a heavy chains are able to fix complement (CDC) and mediate cellular cytotoxicity (ADCC) via interaction with FcR1 on phagocytic mononuclear cells (Burton and Woof, 1992).

```

a

         1          11         21         31

mA33     DIVMTQSQKF MSTSVGDRVS ITCKASQNVR TVVAWYQQKP

hA33     DIQMTQSPSS LSVSVGDRVT ITCKASQNVR TVVAWYQQKP

         41         51         61         71
mA33     GQSPKTLIYL ASNRHTGVPD RFTGSGSGTD FTLTISNVQS
hA33     GL APKTLIYL ASNRHTGVPS RFSGSGSGTD FTFTISSLQP

         81         91         101      108
mA33     EDLADYFCLQ HWSYPLTFGS GTKLEVKR
hA33     EDIATYFCQQ HWSYPLTFGQ GTKVEVKR

b
         1          11         21         31
mA33     EVKLVESGGG LVKPGGSLKL SCAASGFAFS TYDMSWVRQT
hA33     EVQLLESGGG LVQPGGSLRL SCAASGFAFS TYDMSWVRQA

         41         51 52a      59 60       70
mA33     PEKRLEWVAT ISSGGSYTYY LDSVKGRFTI SRDSARNTLY
hA33     PGKGLEWVAT ISSGGSYTYY LDSVKGRFTI SRDSSKNTLY

         80  82a-82c 86 87       97         107    113
mA33     LQMSSLRSED TALYYCAPTT VVPFAYWGQG TLVTVSA
hA33     LQMNSLQAED SAIYYCAPTT VVPFAYWGQG TLVTVSS
```

**Figure 2** Amino acid sequences of the light (**a**) and heavy (**b**) chain variable domains of the murine and humanised A33 antibodies. The sequence of the mouse antibody as deduced from cDNA (mA33) is shown aligned with the humanised antibody sequence (hA33). The humanised framework sequence is derived from the human antibody LAY (Adair *et al.*, 1992). The three complementarity-determining regions in each chain are underlined. Residues in the LAY framework that have been replaced with mouse A33 sequence are double underlined. Numbering as described by Kabat *et al.* (1987).

The murine variable regions of A33 were humanised according to the strategy described by Adair *et al.* (1991). This strategy involves using as frameworks heavy and light chains with the greatest overall homology to the murine antibody, and transferring into these frameworks all the residues from the murine antibody predicted to be involved in antigen binding. The $V_H$ of A33 shows closest homology (70%) to the consensus sequence of human subgroup $V_H$III, while the $V_L$ shows greatest homology to the consensus sequence of human subgroups $V_L$I and $V_L$IV (62%). From these subgroups LAY, which has a $V_H$III heavy chain and $V_L$I light chain, was chosen as the human framework. Figure 2 shows the amino acid sequences of the humanised light and heavy chains. For the light chain residues 1–23, 35–45, 47–49, 57–86, 88 and 98–108 inclusive were derived from the LAY sequence, (numbering as in Kabat *et al.*, 1987) and the residues 24–34, 46, 50–56, 87 and 89–97 inclusive were derived from the murine sequence. Residues 24–34, 50–56 and 89–97 correspond to the complementarity determining regions (CDRs, Kabat *et al.*, 1987). Residues 46 and 87 are predicted to be at the interface of the light and heavy variable regions. Residue 46 is usually a leucine in human antibody sequences and residue 87 is usually either a phenylalanine or tyrosine.

For the heavy chain, residues 2–26, 36–49, 66–71, 74–82a, 82c–85, 87–93 and 103–113 inclusive were derived from the LAY sequence while residues 1, 27–35, 50–65, 72, 73, 82b, 86 and 94–102 inclusive were derived from the murine sequence. Residues 31–35, 50–65 and 95–102 in the heavy chain correspond to the CDRs. The murine-derived amino acids in the framework regions were included for the following reasons. Residue 1 is usually solvent accessible and in the vicinity of the CDR region (residues 27–30) LAY has a residue, alanine, not normally found at this position in human or murine $V_H$ sequences and therefore the murine residue was used. At positons 72 and 73 the murine residue was used because of the predicted proximity to CDR2 and also, in the case of residue 72, to remove the possibility of introducing an *N*-linked glycosylation site into the variable domain by the use of the LAY framework (see also Co *et al.*, 1991). The murine sequence was also used at the inter-domain residue 94, where A33 has a proline, not normally found at this position. Murine residues were used at positions 82 and 86 because the use of the human amino acids at these positions in a humanised antibody with LAY frameworks has previously been found to be deleterious for the expression of the heavy chain (Adair *et al.*, 1992).

**Figure 3** SDS polyacrylamide gel of humanised A33 IgG and TFM under non-reducing conditions. Lane 1, hA33 IgG; lane 2, hA33 Fab'Δcys; lane 3, hA33 Fab'Δcys cross-linking mix; lane 4, purified hA33 TFM.

### *Preparation and* in vitro *characterisation of hA33 IgG1 and TFM*

Small amounts of the humanised antibody were produced in a transient expression system in CHO cells to establish that it bound SW1222 cells expressing the antigen. Stable NS0 cell lines were then isolated to produce larger quantities of purified material, for both hA33 IgG and Fab'Δcys. The best cell lines produced approximately 700 mg $l^{-1}$ hA33IgG1 and 500 mg $l^{-1}$ Fab'Δcys in suspension culture.

Figure 3 shows SDS–PAGE analysis of the purified antibodies under non-reducing conditions. It demonstrates that the purified hIgG was homogeneous and fully assembled. High-performance liquid chromatography (HPLC) analysis also demonstrated it was free of aggregates (data not shown). As expected the hFab'Δcys was recovered largely in the form of monovalent Fab' with little in the form of $F(ab')_2$, consistent with results for other recombinant Fab' fragments (King *et al.*, 1992, 1994). Cross-linking of Fab'Δcys to TFM was achieved with a yield of 60–65%, as shown by HPLC analysis in Figure 4. SDS–PAGE analysis of the purified TFM (Figure 3) showed a single species of approximately 150 kDa under non-reducing conditions.

Figure 5 shows Scatchard analysis for the murine antibody and hIgG1 binding to SW1222 cells. These data suggest both antibody forms have equilibrium dissociation constants ($K_D$s) of 1.3 nM and have approximately 300 000 sites per cell. The antigen-binding activity of hTFM was compared with those of monovalent hFab'Δcys and hIgG in competition binding assays in which these species were asked to compete with murine IgG for binding to Colo205 cells expressing the antigen. The results (Figure 6) demonstrate that the monovalent Fab' fragment binds less well than the bivalent IgG as expected. The trivalent hTFM, on the other hand, showed approximately 2-fold better binding than hIgG, presumably as a result of increased avidity due to the extra antigen binding site. This finding is consistent with results for chimeric B72.3, for which TFM also showed 2- to 3-fold better binding to antigen than IgG (King *et al.*, 1994).

**Figure 4** HPLC profiles at 280 nm of (**a**) hA33 Fab′, (**b**) hA33 Fab′ after cross-linking to TFM and (**c**) purified hA33 TFM. HPLC gel filtration was carried out on a DuPont Zorbax GF-250 column run at 1 ml $min^{-1}$ in 0.2 M sodium phosphate buffer pH 7.0. (**a**) hA33 Fab′ peak has a retention time of 10.5 min with a minor peak of $F(ab')_2$ at 9.7 min and a buffer peak at 12.2 min. (**b**) After cross-linking the major peak represents TFM at 9.0 min. Minor peaks represent di-Fab at 9.6 min, residual monomeric Fab′ at 10.5 min, a small amount of aggregate at 8.2 min and the same buffer peak at 12.2 min. (**c**) After purification the TFM peak is still seen at 9.0 min, the only other visible peak being the buffer peak at 12.2 min.

**Figure 5** Scatchard plot of humanised A33 (Δ) and mouse A33 (O) binding to SW1222 cells. Experimental details as in Materials and methods. $K_D$ values of 1.28 nM for murine A33 IgG and 1.27 nM for humanised A33 IgG were calculated from linear regression analysis of the data points.

*Biodistribution and pharmacokinetics of hA33 IgG1, DFM and TFM*

Immunoconjugates of hIgG were prepared for $^{90}Y$ labelling by derivatisation with the 12N4-maleimide linker CT77. An average macrocycle loading of 1.2 per molecule was achieved, and the immunoconjugate was shown to be fully immunoreactive using the competition-based FACs assay both before and after radiolabelling (data not shown). For biodistribution experiments, radiolabelling was achieved to a sp. act. of 2 μCi $\mu g^{-1}$ with >95% incorporation of $^{90}Y$.

**Figure 6** Competitive binding assay for hA33 Fab′Δcys, IgG and TFM binding to Colo205 cells. hA33 IgG (Δ), TFM (+), DFM (□), and Fab′Δcys (●) were competed with FITC-labelled murine A33 IgG and results (mean of triplicate determinations) plotted as fluorescence units *vs* nM binding sites.

**Figure 7** Time course study showing biodistribution of $^{90}Y$-labelled humanised A33 in nude mice bearing SW1222 tumour xenografts. Mice were injected i.v. with 19 μCi (10 μg) of $^{90}Y$-labelled hA33 each. Groups of four mice were killed at 3 (■), 24 (□), 48 (▩) and 144 h (▨) post-administration and the amount of activity was determined in tumour and normal tissues. Each column represents the mean obtained from four mice, error bars represent the standard deviation of the mean.

Figure 7 shows the biodistribution of $^{90}Y$-labelled hA33 IgG at 3, 24, 48 and 144 h in mice bearing subcutaneous SW1222 xenografts, with tissue uptake plotted as per cent injected dose per gram of tissue. In general, a favourable biodistribution was achieved, with high levels of activity localised to the tumour and little or no accumulation in any normal tissue. The biodistribution of the humanised A33 immunoconjugate was not significantly different from that of the murine antibody in the same xenograft system at these time points (Antoniw *et al.*, manuscript in preparation). For both the murine and humanised antibodies the level of activity localised to the tumour increased with time, even though levels in all other tissues were falling, which led to increasing tumour to normal tissue ratios over time (Figure 8). To assess whether this was a feature of the antibody itself or the radioisotope used, a biodistribution experiment was also carried out with humanised A33 labelled with $^{125}I$. In this experiment the absolute levels of isotope retained by the tumour were slightly lower but the tumour to blood ratios were very similar, suggesting that the increasing localisation is a property of the A33 antibody rather than the nature of the isotope/chelator system. The lower absolute levels of $^{125}I$ labelled hA33 localised to the tumour are probably the result

**Figure 8** Tumour to normal tissues ratio of injected dose per gram at different time intervals after administration. Mice bearing SW1222 tumour xenograft were injected i.v. with either (**a**) 19 $\mu$Ci (10 $\mu$g) $^{90}$Y-labelled hA33 or (**b**) 8 $\mu$Ci (10 $\mu$g) $^{125}$I-labelled hA33. Groups of mice were killed at each time point and the amount of activity was determined in tumour and normal tissues. Data are expressed as the mean value with error bars denoting standard deviation of the mean ($n = 4$). The value for muscle with $^{90}$Y-labelled hA33 at 144 h is $127 \pm 38$. ■, 3 h; ▨, 24 h; □, 48 h; ▩, 168 h.

of dehalogenation of the radioiodinated antibody (Brown *et al.*, 1987).

A series of experiments was performed to compare the biodistribution and pharmacokinetics of hA33 IgG, DFM and TFM. It was consistently observed that humanised TFM and all other humanised fragments examined clear aberrantly quickly from the circulation of mice, far more quickly than the equivalent murine fragments. This phenomenon was shown to be specific to mice and did not occur in rats, guinea pigs or monkeys (data not shown). Figure 9 shows a comparison of biodistribution for hA33 IgG, DFM and TFM in guinea pigs. It demonstrates the more rapid blood clearance of the DFM and TFM, with blood activities falling to 0.01 and 0.02% i.d. g$^{-1}$ respectively at the 144 h time point. Blood activity for hIgG was much higher, at 0.4% i.d. g$^{-1}$, at this time point. Figure 9 demonstrates very clearly that for DFM much higher levels of radioactivity are taken up by the kidney than for IgG and TFM. This high activity for the DFM clears much more slowly from the kidney than from the blood. At early time points kidney levels were a little higher for TFM than IgG but much lower than for DFM, and the activity cleared much faster from the kidney for TFM than for DFM. These results for A33 are consistent with the view that the kidney is the major organ of clearance for TFM.

The pharmacokinetics of hA33 IgG, TFM and DFM were compared in cynomolgus monkeys. Owing to safety considerations, these components were labelled with $^{111}$In rather than $^{90}$Y, previous work having suggested that $^{90}$Y-labelled IgG and fragments show pharmacokinetics and biodistribution very similar to those labelled with $^{111}$In (data not shown). The plasma clearance profiles are shown in Figure 10 with the alpha and beta phase half-life values in Table I. As expected from pharmacokinetic data in mice and guinea pigs both TFM and DFM cleared faster from the circulation than IgG. In addition, DFM cleared more quickly than TFM. When plasma clearance was examined without decay correction for the isotope (Table II) the data was most consistent with monophasic kinetics for IgG and TFM, with biphasic kinetics for DFM. Dosimetric calculations based on these data suggest that when labelled with $^{90}$Y at equivalent amounts of radioactivity injected, the DFM would give approximately a 5-fold lower absorbed dose to the bone marrow than IgG, while TFM would give a 2-fold lower absorbed dose (Table II).

### Discussion

In general, the antibodies directed to tumour-associated antigens that are currently available have been produced from rodent hybridomas and are immunogenic in man. This has been a major obstacle to radioimmunotherapy, since although the first doses of some immunoconjugates have given promising biodistribution and partial therapeutic responses, subsequent doses have been rendered ineffective by rapid clearance due to the HAMA response elicited (Welt *et al.*, 1990; Mountain and Adair, 1992).

We constructed the humanised variant of A33 by substituting into the frameworks of the human antibody LAY all the residues of the murine antibody, which we predict may contribute to antigen binding. These residues comprise the CDRs together with two residues on the light chain and five on the heavy chain which may contribute to the precise positioning of the CDRs for antigen binding (Adair *et al.*, 1991). Scatchard analysis suggested the murine and humanised variants of the antibody have equivalent affinity for the antigen on the surface of colorectal tumour cells.

As yet there are still few data concerning the effectiveness of full humanisation in overcoming the patient immune response to rodent antibodies. In three reports on the immunogenicity of humanised antibodies in monkeys (Hakimi *et al.*, 1991; Singer *et al.*, 1993; Stephens *et al.*, 1994) the immune response was much reduced compared with the parent murine antibodies. In each case, an anti-idiotype response (i.e. directed to the CDRs) developed on repeat dosing. In the case studied in greatest detail (Stephens *et al.*, 1994) the CDRs appear less immunogenic when presented in the human framework since the anti-idiotype response to the humanised antibody was greatly reduced compared with the anti-idiotype component of the immune response to the murine antibody. As yet there are only three reports on the administration of fully humanised antibodies to patients. Two of these studies concerned the humanised antibody CAMPATH-1H. Two non-Hodgkin lymphoma patients treated with 1–20 mg doses of this antibody for up to 43 days showed no anti-CAMPATH-1H response during the course of treatment (Hale *et al.*, 1988). These results should not be over-interpreted, however, because such patients are somewhat immunocompromised before treatment and because the treatment itself is likely to be immunosuppressive. More recently, however, results have become available for eight rheumatoid arthritis patients repeatedly administered with 4–8 mg doses of CAMPATH-1H over 10 days (Isaacs *et al.*, 1992). Significant clinical benefit was seen in seven of the patients, and anti-CAMPATH-1H antibodies were not detectable in any of the patients after this one course of treatment. Of four patients given a second course of treatment, three showed a detectable anti-CAMPATH-1H response. No data are yet available concerning the nature of this response, whether it interferes with efficacy, and if so, whether such interference can be overcome using larger doses. The immune response to the humanised anti-tumour

**Figure 9** Biodistribution of 9 μCi (8 μg) $^{90}$Y-labelled hA33 IgG (■), 13 μCi (10 μg) $^{90}$Y-hA33 TFM (□) and 14 μCi (10 μg) $^{90}$Y-labelled hA33 DFM (▨) in guinea pigs at (**a**) 24 h, (**b**) 48 h, (**c**) 72 h and (**d**) 144 h. Data are expressed as the mean percentage injected dose per gram of tissue with error bars denoting the standard deviation ($n = 4$). The value for DFM in the kidney at 24 h is 9.3 ± 1.6.

**Figure 10** Pharmacokinetic profiles of $^{111}$In-labelled hA33 IgG (■), TFM (○) and DFM (▲) in cynomolgus monkeys. Data were corrected for decay and plotted as mean percentage injected dose remaining at each time point.

**Table I** Mean plasma clearance half-life values for $^{111}$In-labelled hA33 IgG, TFM and DFM in groups of two cynomolgus monkeys (decay corrected)

| *Antibody form* | $t_{1/2\alpha}$*(h)* | $t_{1/2\beta}$ *(h)* |
|---|---|---|
| IgG | 15.8 | 129.0 |
| TFM | 12.8 | 53.7 |
| DFM | 10.8 | 42.0 |

Values were obtained using a two-compartment model (SIPHAR).

**Table II** Pharmacokinetics of $^{111}$In-labelled hA33 IgG, TFM and DFM in cynomolgus monkeys

| *Antibody form* | *Effective half-life (h)* | *Absorbed dose to red marrow and endosteum (rad mCi$^{-1}$)* |
|---|---|---|
| IgG | 40.5 | 12.0 |
| TFM | 21.9 | 6.3 |
| DFM | 4.7α/23.6β | 2.6 |

Values for effective half-life and estimates of absorbed dose in red marrow and endosteum in man for $^{90}$Y-labelled antibodies were determined as described in Materials and methods.

necrosis factor antibody CDP571 has been examined after administration of single doses of 0.1–10 mg kg$^{-1}$ to human volunteers (Stephens *et al.*, 1994). Administration of the lower doses led to the development of a weak anti-idiotype response, predominantly of the IgM isotype. Anti-CDP571 antibodies were very low or undetectable after administration of the higher doses. The limited clinical data available for fully humanised antibodies therefore suggest they will show substantially longer half-lives and greatly reduced immunogenicity compared with murine antibodies.

As described previously (Harrison *et al.*, 1991) the 12N4 macrocycle gives extremely stable chelation of $^{90}$Y, with no significant escape of the isotope *in vivo*. The potential immunogenicity of the macrocycle, however, as well as that of the humanised antibody carrier is a significant issue. It is far from clear whether macrocyclic chelators are likely to be immunogenic in patients. Kosmas *et al.* (1992) reported the rapid development of an anti-macrocycle immune response in ovarian cancer patients administered with antibody conjugates carrying $^{90}$Y or $^{111}$In in the macrocycle *p*-nitrobenzyl-DOTA. Curiously, administration conditions favouring immunogenicity (i.p. injection of relatively large doses with a high proportion of aggregates) led to a stronger immune response to the macrocycle than to the antibody carrier, and some of these patients manifested symptoms of serum sickness.

More recently Watanabe *et al.* (1994) have examined the immunogenicity of a similar chelator in rabbits, and concluded that an anti-macrocycle response develops in animals injected with the macrocycle conjugated to immunogenic carrier proteins such as murine antibodies, but not in those injected with rabbit antibody conjugates. Studies by Kosmas *et al.* (1992) to characterise the anti-macrocycle response in patients concluded that it is predominantly directed to the DOTA ring structure itself rather than to the linker, which contains an aromatic ring. We have conducted a study to examine the immunogenicity of the 12N4 macrocycle in mice. The only major difference between the chelator used in these studies and that used by Kosmas *et al.* (1992) is the presence of the aromatic ring on the latter. The results (TS Baker *et al.*, manuscript in preparation) were similar to those of Watanabe *et al.* (1994), in that no anti-macrocycle response could be detected in mice injected with the macrocycle conjugated to a mouse antibody even using conditions very favourable to effective immunisation. Mice injected with the macrocycle conjugated to an immunogenic protein such as a chimeric antibody developed an immune response with components directed to both the carrier protein and the macrocycle. We conclude it is unlikely that immunogenicity of the 12N4 macrocycle will prove to limit the effectiveness of humanised antibodies carrying $^{90}Y$ administered at reasonable doses intravenously.

Uptake of the $^{90}Y$-labelled humanised antibody by human colorectal xenografts in nude mice was similar to that reported for murine A33 labelled with $^{90}Y$ via the same methodology (P Antoniw *et al.*, manuscript in preparation). This high tumour uptake augurs well for the prospects of achieving therapeutically effective radiation doses in colorectal cancer patients. $^{90}Y$ continued to accumulate in the tumour for the humanised antibody throughout the 144 h after injection over which measurements were made. This continued accumulation was also observed for hA33 with both of the other colorectal tumour cell lines studied (P Antoniw *et al.*, unpublished). hA33 labelled with $^{125}I$ shows less accumulation, and the increased retention of $^{90}Y$ by the tumour cells may result from the inability of the macrocyclic chelator to egress from the cells after internalisation.

Clinical studies with chimeric and humanised antibodies have also suggested that these antibodies have a substantially longer circulating half-life than murine antibodies. Humanised antibodies for radioimmunotherapy may therefore deliver an increased dose to the bone marrow from the circulating conjugate, which would give decreased therapeutic ratios unless tumour uptake is correspondingly increased. The production of TFM is an attempt to circumvent this problem. The binding avidity of TFM is increased compared with IgG and pharmacokinetic studies show faster blood clearance in both guinea pigs and cynomolgus monkeys. The mean beta-phase half-life of the IgG1 in cynomolgus monkeys (129 h) is in the range previously observed for humanised antibodies in this species (Hakimi *et al.*, 1991; Singer *et al.*, 1993; Stephens *et al.*, 1994). The mean beta-phase half-life for TFM in cynomolgus monkeys (53.7 h) is less than half that of IgG, and dosimetric calculations indicate that for an equivalent injected dose of $^{90}Y$ this shorter half-life would give an approximately 2-fold benefit for TFM in terms of the radiation dose to the bone marrow. Since the latter is usually the dose-limiting toxicity in clinical studies with therapeutic radioimmunoconjugates, it is likely TFM would allow a higher maximum tolerated dose.

Unlike all other antibody fragments carrying metallic isotopes which have been examined (see for example Sharkey *et al.*, 1990) the TFM does not lead to high-level accumulation of the isotope in the kidney or other non-specific tissues. In the present studies DFM showed faster blood clearance and projected bone marrow doses lower than those for both IgG and TFM, but is clearly not an acceptable vehicle for delivering $^{90}Y$ because it leads to unacceptably high accumulation of this isotope in the kidney. Such kidney accumulation does not occur for DFM labelled with radioiodine (data not shown), and it is likely that the DFM is the most appropriate delivery vehicle for therapy with $^{131}I$.

Clinical success with radioimmunotherapy has so far largely been restricted to haematological malignancies such as lymphomas and leukaemias. hA33 TFM carrying $^{90}Y$ in the 12N4 macrocycle represents one of the first attempts to optimise all components of a radioimmunoconjugate for treatment of solid tumours. Evaluation of this second generation immunoconjugate in colorectal cancer patients may reveal new potential for radioimmunotherapy of solid tumours. As the first stage in clinical evaluation of this technology, a quantitative biodistribution study in colorectal cancer patients using hA33 TFM carrying $^{111}In$ is in progress.

**Acknowledgements**

At Celltech, we thank A Millican, K Millar and B Boyce for synthesis of CT77, G Roberts and A Campbell for assistance in cloning the A33 variable region genes and J Turner and J Scothern for synthesis of oligonucleotides. We also thank P Jupp of American Cyanamid, Gosport, UK, for synthesis of the cross-linker CT998.

### References

ADAIR JR, ATHWAL DS AND EMTAGE JS. (1991). World patent application WO91/09967.

ADAIR JR, BODMER MW, MOUNTAIN A AND OWENS RJ. (1992). World patent application WO92/010509.

BAKER TS, BEGENT RHJ, DEWJI MR, CONLAN J AND SECHER DS. (1991). Characterization of the antibody response in patients undergoing radioimmunotherapy with chimeric B72.3. *Antibody Immunoconj. Radiopharmacol.*, **4**, 799–809.

BEBBINGTON CR, RENNER G, THOMSON S, KING D, ABRAMS D AND YARRANTON GT. (1992). High-level expression of a recombinant antibody from myeloma cells using a glutamine synthetase gene as an amplifiable selectable marker. *Bio/Technology*, **10**, 169–175.

BROWN BA, COMEAU RD, JONES PL, LIBERATORE FA, NEACY WP, SANDS H AND GALLAGHER BM. (1987). Pharmacokinetics of the monoclonal antibody B72.3 and its fragments labelled with either $^{125}I$ or $^{111}In$. *Cancer Res.*, **47**, 1149–1154.

BURTON D AND WOOF JM. (1992). Human antibody effector function. *Immunology*, **1**, 1–84.

CARON PC, CO MS, BULL MK, AVDALOVIC NM, QUEEN C AND SCHEINBERG DA. (1992). Biological and immunological features of humanized M195 (anti-CD33) monoclonal antibodies. *Cancer Res.*, **52**, 6761–6767.

CO MS, DESCHAMPS M, WHITLEY RJ AND QUEEN C. (1991). Humanized antibodies for antiviral therapy. *Proc. Natl Acad. Sci. USA*, **88**, 2869–2873.

COCKETT MIC, BEBBINGTON CR AND YARRANTON GT. (1990). High-level expression of tissue inhibitor of metalloproteinases in Chinese hamster ovary cells using glutamine synthetase gene amplification. *Bio/Technology*, **8**, 662–667.

DAUGHERTY BL, DEMARTINO JA, LAW M-F, KAWKA DW, SINGER II AND MARK GE. (1991). Polymerase chain reaction facilitates the cloning, CDR-grafting and rapid expression of a murine monoclonal antibody directed against the CD18 component of leukocyte integrins. *Nucleic Acids Res.*, **19**, 2471–2476.

DENARDO GL, KROGER LA, DENARDO SJ, MIERS LA, SALAKO Q, KUKIS DL, FAND I, SHEN S, RENN O AND MEARES CF. (1994). Comparative toxicity studies of yttrium-90 MX-DTPA and 2-IT-BAD conjugated monoclonal antibody (BrE-3). *Cancer*, **73**, Suppl. 1012–1022.

DESHPANDE SV, DENARDO SJ, KUKIS DL, MOI MK, MCCALL MJ, DENARDO GL AND MEARES CF. (1990). Yttrium-90 labelled monoclonal antibody for therapy: labelling by a new macrocyclic bifunctional chelating agent. *J. Nucl. Med.*, **31**, 473–479.

HAKIMI J, CHIZZONITE R, LUKE DR, FAMILLETTI PC, BAILON P, KONDAS JA, PILSON RS, LIN P, WEBER DV, SPENCE C, MONDINI LJ, TSIEN W-H, LEVIN JE, GALLATI VH, KORN L, WALDMANN TA, QUEEN C AND BENJAMIN WR. (1991). Reduced immunogenicity and improved pharmacokinetics of humanized anti-Tac in cynomolgus monkeys. *J. Immunol.*, **147**, 1352–1359.

HALE G, DYER MJ, CLARK MR, PHILLIPS JM, MARCUS R, RIECHMANN L, WINTER G AND WALDMANN H. (1988). Remission induction in non-Hodgkin lymphoma with reshaped human monoclonal antibody CAMPATH-1H. *Lancet*, **2**, 1394–1399.

HARRISON A, WALKER C, PARKER D, JANKOWSKI K, COX J, CRAIG A, SANSOM J, BEELEY N, BOYCE B, CHAPLIN L, EATON M, FARNSWORTH A, MILLAR K, MILLICAN A, RANDALL A, RHIND S, SECHER D AND TURNER A. (1991). The *In vivo* release of $^{90}$Y from cyclic and acyclic ligand-antibody conjugates. *Nucl. Med. Biol.*, **18**, 469–476.

HNATOWICH DJ, CHINOL M, SIEBACKER DA, GIONET M, GRIFFIN T, DOHERTY PW, HUNTER R AND KASE KR. (1988). Patient biodistribution of intraperitoneally administered 90Y labelled antibody. *J. Nucl. Med.*, **29**, 1428–1434.

ISAACS JD, WATTS RA, HAZLEMAN BL, HALE G, KEOGAN MT, COBBOLD SP AND WALDMANN H. (1992). Humanised monoclonal antibody therapy for rheumatoid arthritis. *Lancet*, **340**, 748–752.

JONES ST AND BENDIG MM. (1991). Rapid PCR-cloning of full length mouse immunoglobulin variable regions. *Bio/Technology*, **9**, 88–89.

KABAT EA, WU TT, REID-MILLER M, PERRY HM AND GOTTESMAN KS. (1987). Sequences of proteins of immunological interest 4th ed. United States Department of Health and Human Services: Washington DC.

KING DJ, ADAIR JR, ANGAL S, LOW DC, PROUDFOOT KA, LLOYD JC, BODMER M AND YARRANTON GT. (1992). Expression, purification and characterisation of a mouse: human chimeric antibody and chimeric Fab' fragment. *Biochem. J.*, **281**, 317–323.

KING DJ, TURNER A, FARNSWORTH APH, ADAIR JR, OWENS RJ, PEDLEY RB, BALDOCK D, PROUDFOOT KA, LAWSON ADG, BEELEY NRA, MILLAR K, MILLICAN TA, BOYCE B, ANTONIW P, MOUNTAIN A, BEGENT RHJ, SHOCHAT D AND YARRANTON GT. (1994). Improved tumour targeting with chemically cross-linked recombinant antibody fragments. *Cancer Res.*, **54**, 6176–6185.

KOSMAS K, SNOOK D, GOODEN CS, COURTENAY-LUCK NJ, MCCALL MJ, MEARES C AND EPENETOS AA. (1992). Development of humoral immune responses against a macrocyclic chelating agent (DOTA) in cancer patients receiving radioimmunoconjugates for imaging and therapy. *Cancer Res.*, **52**, 904–911.

KRAUSE D, SHEARMAN C, LANG W, KANZY EJ AND KURLE R. (1990). Determination of affinities of murine and chimeric anti-(a/b-T cell receptor) antibodies by flow cytometry. *Behring Inst. Mitt.*, **87**, 56–67.

LARSON SM. (1991). Choosing the right radionuclide and antibody for intraperitoneal radioimmunotherapy. *J. Natl Cancer Inst.*, **83**, 1602–1604.

LOBUGLIO AF, WHEELER RH, TRANG J, HAYNES A, ROGERS K, HARVEY EB, SUN L, GHRAYEB J AND KHAZAELI MB. (1989). Mouse/human chimeric monoclonal antibody in man: Kinetics and immune response. *Proc. Natl Acad. Sci. USA*, **86**, 4220–4224.

MOUNTAIN A AND ADAIR JR. (1992). Engineering antibodies for therapy. *Biotechnology and Genetic Engineering Reviews*, **10**, 1–142.

SALEH MN, KHAZAELI MB, WHEELER RH, ALLEN L, TILDEN AB, GRIZZLE W, REISFELD RA, YU AL, GILLIES SD AND LOBUGLIO AF. (1992). Phase I trial of the chimeric anti-GD2 monoclonal antibody ch14.18 in patients with malignant melanoma. *Int. J. Cancer*, **33**, 633–641.

SHARKEY RM, MOTTA-HENNESSY C, PAWLYK D, SIEGAL JA AND GOLDENBERG DM. (1990). Biodistribution and radiation dose estimates for yttrium and iodine labelled monoclonal antibody IgG and fragments in nude mice bearing human colonic tumor xenografts. *Cancer Res.*, **50**, 2330–2336.

SINGER I, KAWKA D, DEMARTINO J, DAUGHERTY B, ELLISTON K, ALVES K, BUSH B, CAMERON PM, CUCA G, DAVIES P, FORREST M, KAZAZIS D, LAW M-F, LENNY A, MACINTYRE D, MEURER R, PADLAN E, PANDY AS, SCHMIDT J, SEAMANS T, SCOTT M, WILLIAMSON A AND MARK G. (1993). Optimal humanization of 1B4, an anti-(CD18) murine monoclonal antibody, is achieved by correct choice of human V-region framework sequences. *J. Immunol.*, **150**, 2844–2857.

STEPHENS P AND COCKETT M. (1989). The construction of a highly efficient and versatile set of mammalian expression vectors. *Nucleic Acids Res.*, **17**, 7110.

STEPHENS S, ATHWAL D, CHAPLIN LC, EMTAGE JS, SOPWITH M, TEMPLE G, VETTERLEIN O AND BODMER M. (1994). Pharmacokinetics of CDP571, an anti-TNFα engineered human antibody in cynomolgous monkey and man. *Circ. Shock Suppl.* **1**, 60.

STEWART JS, HIRD V, SNOOK D, DHOKIA B, SIVOLAPENKO GB, HOOKER G, TAYLOR-PAPADIMITRIOU J, ROWLINSON G, SULLIVAN M, LAMBERT HE, COULTER C, MASON WP, SOUTTER WP AND EPENETOS AA. (1990). Intraperitoneal Yttrium-90 labelled monoclonal antibody in ovarian cancer. *J. Clin. Oncol.*, **8**, 1941–1950.

TURNER A, KING DJ, FARNSWORTH APH, RHIND SK, PEDLEY RB, BODEN J, BODEN R, MILLICAN TA, MILLAR K, BOYCE B, BEELEY NRA, EATON MAW AND PARKER D. (1994). Comparative biodistribution of indium-111-labelled macrocycle chimeric B72.3 antibody conjugates in tumour-bearing mice. *Br. J. Cancer*, **70**, 35–41.

WATANABE N, GOODWIN DA, MEARES CF, MCTIGUE M, CHAOVAPONG W, RANSONE CM AND RENN O. (1994). Immunogenicity in rabbits and mice of an antibody-chelate conjugate: comparison of (S) and (R) macrocyclic enantiomers and an acyclic chelating agent. *Cancer Res.*, **54**, 1049–1054.

WELT S, DIVGI CR, REAL FX, YEH SD, PILAR GC, FINSTAD CL, SAKAMOTO J, COHEN A, SIGURDSON ER, KEMENY N, CARSWELL EA, OETTGEN HF AND OLD LJ. (1990). Quantitative analysis of antibody localization in human metastatic colon cancer: Studies with monoclonal antibody A33. *J. Clin. Oncol.*, **8**, 1894–1896.

WELT S, DIVGI CR, KEMENY N, FINN RD, SCOTT AM, GRAHAM M, GERMAIN JS, CARSWELL-RICHARDS E, LARSON SM, OETTGEN HF AND OLD LJ. (1994). Phase 1/11 study of iodine-131-labelled monoclonal antibody A33 in patients with advanced colon cancer. *J. Clin. Oncol.*, **12**, 1561–1571.

WHITTLE N, ADAIR J, LLOYD C, JENKINS L, DEVINE J, SCHLOM J, RAUBITSHEK A, COLCHER D AND BODMER M. (1987). Expression in COS cells of a mouse/human chimaeric B72.3 antibody. *Protein Engineering*, **1**, 499–505.

WHITWELL JR AND SPIERS FW. (1976). Calculated beta-ray dose factors for trabecular bone. *Phys. Med. Biol.*, **27**, 16–38.